# Occurrence of gastrointestinal parasites in dogs in a rural area of Santa Catarina, Brazil

**DOI:** 10.1590/S1984-29612023061

**Published:** 2023-10-13

**Authors:** Alisson Andrade Arruda, Katia Denise Saraiva Bresciani, Simone Silmara Werner, Bruna Fernanda da Silva

**Affiliations:** 1 Programa de Pós-Graduação em Ambiente e Saúde, Universidade do Planalto Catarinense - UNIPLAC, Lages, SC, Brasil; 2 Departamento de Produção e Saúde Animal, Universidade Estadual Paulista - FMVA/UNESP, Araçatuba, SP, Brasil; 3 Departamento de Informática e Estatística - INE-CTC, Universidade Federal de Santa Catarina - UFSC, Florianópolis, SC, Brasil

**Keywords:** Dogs, helminths, Protozoa, microscopy, zoonoses, public health, Caninos, helmintos, Protozoários, microscopia, zoonoses, saúde pública

## Abstract

We investigated the occurrence of gastrointestinal parasites in dogs in rural areas of the municipality of Painel, Santa Catarina, Brazil. For this, 91 canine feces samples were collected from 82 farms between August, 2017 and January, 2018. These fecal materials were processed using the techniques of spontaneous sedimentation, centrifugation-floatation in zinc sulfate and Ziehl-Neelsen staining. A questionnaire in the form of an interview was administered to the dogs’ owner and information about the farm and the main care provided for the dogs was obtained. Among 91 sampled dogs, 27 (29.7%) were positive for at least one parasite species. *Ancylostoma* was the most prevalent genus (16.5%), followed by *Giardia duodenalis* (14.3%), *Trichuris vulpis* (6.6%), *Toxocara canis* (5.5%), *Entamoeba* spp. (4.8%), *Cryptosporidium* spp. (3.3%) and Taeniidae (1.1%). Most dogs do not receive veterinarian care and rarely received antiparasitic treatment. They were free to roam and had free access to animal remains and garbage, which was reflected in the significant associations with the occurrence of parasites that were found. We conclude that rural dogs harbor gastrointestinal parasites, but that their owners are unaware of the risks that these parasites can bring to human health.

## Resumo

Foi investigada a ocorrência de parasitos gastrintestinais com potencial zoonótico, em cães de área rural do munícipio de Painel, Santa Catarina, Brasil. Para isso, foram colhidas amostras de fezes de 91 cães de 82 propriedades rurais, no período de agosto de 2017 a janeiro de 2018. Este material fecal foi processado pelas técnicas de sedimentação espontânea, centrífugo-flutuação em sulfato de zinco e coloração pelo Ziehl-Neelsen. Um questionário em forma de entrevista foi aplicado aos tutores dos cães e foram obtidas informações sobre a propriedade rural e principais cuidados com os cães. Entre as 91 amostras de fezes, em 27 (29,7%) foi detectada a presença de parasitos gastrintestinais, sendo *Ancylostoma* o gênero mais prevalente (15 / 16,5%), seguido por *Giardia duodenalis* (13 / 14,3%), *Trichuris vulpis* (6 / 6,6%), *Toxocara canis* (5 / 5,5%), *Entamoeba* spp. (4 / 4,8%), *Cryptosporidium* spp. (3 / 3,3%) e Taeniidae (1 / 1,1%). A maioria dos cães não recebia cuidados veterinários ou antiparasitários. Concluiu-se que os cães de área rural albergam parasitos gastrintestinais, porém seus tutores desconhecem os riscos que esses parasitos podem trazer para a saúde humana.

## Introduction

Despite the physiological and psychological benefits that the relationship with pets brings to humans, there is an inherent risk of transmission of diseases to their owners (Overgaauw et al., 2009). Apparently, healthy dogs can host and excrete zoonotic parasites that are harmful to human health and domestic livestock (Sterneberg-Van Der Maaten et al., 2016).

With regard to public health, dogs play a major role in the transmission of potentially zoonotic parasites. Among these, the following can be highlighted: the nematodes *Toxocara canis*, *Trichuris vulpis*, *Ancylostoma caninum*, *Ancylostoma braziliense* and *Strongyloides stercoralis*; the cestodes *Echinococcus granulosus* and *Dipylidium caninum*; and the protozoa *Entamoeba* spp., *Giardia duodenalis* and *Cryptosporidium* spp. (Robertson & Thompson, 2002; Dantas-Torres & Otranto, 2014).

These infections are transmitted to humans through direct contact with infected dogs or exposure to environments contaminated with infected dog feces or by larvae that can penetrate the skin of the susceptible host (Ezema et al., 2019).

The increasing numbers of dogs raised in homes and rural areas as guard dogs, for companionship, or as working dogs, together with close physical contact with humans, expose humans to parasites and the diseases that they harbor (Sterneberg-Van Der Maaten et al., 2016).

Studies carried out in central and peripheral areas of the city of Lages, Santa Catarina, prove the presence of potentially zoonotic parasites in domiciled and stray dogs (Stalliviere et al., 2013; Quadros et al., 2014), but there are no studies carried out in rural areas of this region.

In this sense, the rural area of Serra Catarinense is widely exploited for rearing ruminants, horses, pigs and agricultural crops. Dogs can be found on almost all farms, as guard dogs or companions, or for the purpose of working with livestock management, which is a common custom in the southern region of Brazil. Therefore, we investigated the occurrence of gastrointestinal parasites in the dog population of rural areas of Santa Catarina, Brazil, and the characteristics of the environment in which these dogs were living, as well as verifying the knowledge of their owners about zoonoses.

## Material and Methods

This study was conducted on 82 farms in the municipality of Painel, state of Santa Catarina, Brazil (27º 55'44” S; 50º 06'18” W; 1,144 m above sea level). Painel has a territorial area of 740.2 km^2^, a population of approximately 2,353 inhabitants, and a human development index (HDI) of 0.664 (IBGE, 2010). The climate is classified as Cfb according to Köppen, with an average annual temperature of 15.3 °C and average annual rainfall of 1543 mm. The municipality is essentially agricultural, with an economy based on agriculture and livestock.

The sample size was obtained considering the population of 502 farms (IBGE, 2006), a 95% confidence level, and a margin of error of 10%, obtaining the number of 81 farms. These farms were then selected according to convenience, but farms were only sampled if they had at least one dog.

Before feces were collected, the dog’s owner read and signed the free and informed consent statement for the inclusion of his/her respective dog in this study. Then, the owner was interviewed through a questionnaire, which asked for information about the characteristics of the farm (use of the land, water source, analysis and treatment, sewage, and garbage destination) as well as some personal information like age, education level, and if had children (1-12 years old) between the farm residents.

The dogs’ owners were also asked about dog management, such as the type of food offered to the dogs (homemade, commercial feed, or mixed), whether their dogs were fed with animal carcass remains, whether they received veterinary care, whether they were dewormed, whether coproparasitological exams had ever been performed on the dogs and whether they had access to the source of water for human consumption or to sewage or garbage.

After this, one sample of feces per dog was collected immediately after inducing evacuation by means of an enema stimulant (monosodium phosphate dihydrate), which was administered rectally. The samples were packed in disposable packaging, in a thermally insulated box for transportation, at a temperature of 2 to 8 °C, and were analyzed within 24 hours.

All fecal samples were subjected to three coproparasitological techniques and evaluated for the presence of parasitic forms. The techniques used were centrifugal flotation on zinc sulfate (ZnSO4), density: 1.18 g/mL (Faust et al., 1938), and spontaneous sedimentation (Hoffman et al., 1934). The diagnosis of parasitic structures was based on the morphological characteristics reported by Soulsby (1987) and Zajac & Conboy (2012). To view *Cryptosporidium* spp. oocysts, thin smears were placed on glass slides and subsequently stained using the acid-resistant technique of Ziehl-Neelsen (Vohra et al., 2012; Adeyemo et al., 2018).

### Statistical analysis

The descriptive analysis of data relating to the characteristics of the farms and animals and the prevalence of the parasites found in the dogs’ feces was performed using the IBM SPSS software (Statistical Package for the Social Sciences), version 20.

The logistic binomial model was fitted to investigate the parasite presence/absence. The characteristics of the farms and their dogs were obtained through the questionnaire and were considered explanatory variables in the model. The variables selection was performed through a stepwise method using the Akaike information criterion (AIC) (Venables & Ripley, 2002). The analyses were performed with R environment (R Core Team, 2017).

## Results

The average age of the dogs’ owner was 41 years (SD ± 12.9 years), with a range from 18 to 74 years. Regarding education, one was illiterate (1.2%), and 32 (39.0%), 33 (40.2%) and 16 (19.5%) had attended elementary, high school and higher education, respectively. The residents on 26 farms (31.7%) included children between the ages of 1-12 years.

Among the 82 farms visited, livestock rearing was the main activity (87.8%). The water source was mainly from spring/river (92.7%), that no received treatment or analysis of its quality (97.6%) (Table 1). The sewage destination was mainly in septic tanks (78%) and the garbage was collected by municipal garbage collection in 69.5% of the farms. However, the sewage was also dumped into the river (20.8%) and the garbage burned or discharged into the environment (30.55) (Table 1).

**Table 1:** Absolute and percentage frequencies of characteristics of the farm in a rural area of Santa Catarina, Brazil.

**Variables and categories**	**Number**	**%**
**Use of the land**		
Livestock rearing	72	87.8
Other activities*	10	12.2
**Water source**		
Spring/river	76	92.7
Artesian well	4	4.9
Treatment station	2	2.4
**Water quality analysis**		
Yes	2	2.4
Not	80	97.6
**Water treatment**		
Yes	2	2.4
Not	80	97.6
**Sewage destination**		
Septic tank	64	78.0
Dumped into river	17	20.8
Discharged in the environment	1	1.2
**Garbage destination**		
Garbage collection	57	69.5
Burned or discharged in the environment	25	30.5

* Orchard, horticulture, reforestation, and rural tourism.

All farms visited had at least one dog, being that 73 had one dog (89.0%), six had two dogs (7.3%) and three had three dogs (3.7%). Then, the sample was formed by 91 dogs mainly composed of male dogs (67%), adults (78%), and pure breeds (74.7%), with the function of assisting their owners in livestock management (62.6%) (Table 2). The mean age of the dogs was three years (SD ± 1.8 years); the youngest was two months and the oldest was nine years.

**Table 2:** Absolute and percentage frequencies of characteristics and management of dogs in a rural area of Santa Catarina, Brazil.

**Variables and categories**	**Number**	**%**
**Gender**		
Male	61	67.0
Female	30	33.0
**Age**		
Puppy (≤ 6 months old)	4	4.4
Young (6 to 12 months old)	16	17.6
Adult (≥ 13 months old)	71	78.0
**Breed**		
Purebreds*	68	74.7
Crossbred	23	25.3
**Function**		
Cattle management	57	62.6
Hunting, companionship and guarding	34	37.4
**Food and water usage**		
Mixed type (composed of homemade food and/or commercial feed)	80	87.9
Commercial feed	8	8.8
Homemade food	3	3.3
Free access to carcasses or remains of animals slaughtered on the farm	75	82.4
Free access to the human and animal water source	86	94.5
Free access to a garbage	29	31.9
**Veterinary care**		
Yes	4	4.4
No	87	95.6
**Coproparasitological examinations**		
Yes	0	0
No	91	100
**Antiparasitic use**		
One dose during the lifetime	69	75.8
Never	22	24.2

* Dog purebreds are composed mainly of blue heelers, border collies, collies, and German shepherds.

The food that these dogs received was predominantly of mixed type, i.e., composed of homemade food and/or commercial feed (87.9%) and 82.4% of them had free access to carcasses or remains of animals slaughtered on the farm itself (Table 2). All the dogs in this study were free to roam around the farm and most had access to the human and animal water source (Table 2).

Only four dogs receive regular veterinary care, coproparasitological examinations were never performed on these dogs and regarding the use of anthelmintics, the owners reported that 75.8% of the dogs had received at least one dose of antiparasitic during their lifetime (Table 2).

**Figure 1:**
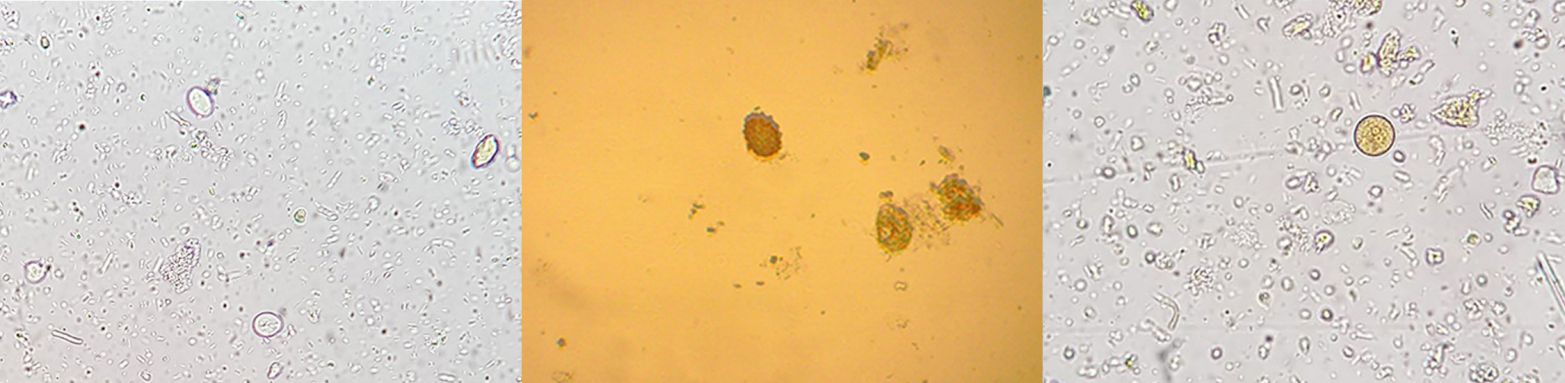
Cysts and eggs from gastrointestinal parasites in fecal samples from dogs in a rural area of Santa Catarina, Brazil. (A) *Giardia duodenalis* (B) *Toxocara canis* and (C) *Entamoeba* spp.

Out of the total of 91 canine fecal samples examined in this study, 27 (29.7%) harbored gastrointestinal parasites (Table 3). Among the positive animals, *Ancylostoma* spp. and *G. duodenalis* (Figure 1A) were the main species, followed by *T. vulpis*, *T. canis* (Figure 1B), *Entamoeba* spp. (Figure 1C), *Cryptosporidium* spp. and Taenidae.

**Table 3:** Absolute and percentage frequencies of gastrointestinal parasites diagnosed in dogs in a rural area of Santa Catarina, Brazil, and the occurrence of polyparasitism.

**Gastrointestinal parasites**	**Number**	**%**
*Ancylostoma* spp.	15	16.5
*Giardia duodenalis*	13	14.3
*Trichuris vulpis*	6	6.6
*Toxocara canis*	5	5.5
*Entamoeba* spp.	4	4.8
*Cryptosporidium* spp.	3	3.3
Taeniidae	1	1.1
Total positive samples	27	29.7
**Mixed infections**		
*Ancylostoma* + *G. duodenalis*	2	2.2
*Ancylostoma* + *Entamoeba* spp.	1	1.1
*Ancylostoma* + *T. vulpis*	1	1.1
*G. duodenalis* + *Entamoeba* spp.	1	1.1
*Ancylostoma* + *G. duodenalis* + *Entamoeba* spp.	1	1.1
*Ancylostoma* + *G. duodenalis* + *Cryptosporidium* spp.	1	1.1
*Ancylostoma* + *T. vulpis + T. canis* + Taeniidae	1	1.1
*Ancylostoma* + *T. vulpis + G. duodenalis* + *T. canis* + *Cryptosporidium* spp.	1	1.1
*Ancylostoma* + *T. vulpis + G. duodenalis* + *T. canis + Cryptosporidium* spp. + *Entamoeba* spp.	1	1.1

Among the 27 parasitized dogs, polyparasitism was observed in 10 dogs (10.9%), of which five were parasitized by two different species, two by three species, one by four species, one by five species and one by six species (Table 3).

When asked about which diseases dogs are able to transmit to humans, most of them responded rabies (45 / 54.9%), four (4.9%) mentioned worms, three (3.7%) responded allergies, *Leishmania* spp., and fleas; two (2.4%) ticks, one (1.2%) mange, fungi and infections. On the other hand, 19 (23.2%) said that dogs do not transmit diseases to humans.

Regarding participation in campaigns or actions for controlling zoonosis, all the participants reported that they had never participated in such actions.

According to the model that was fitted to describe the occurrence of gastrointestinal parasitic infection in dogs, regardless of species, factors such as the tutor’s education level (p = 0.0026) were significant, as well the age (p < 0.0001), gender (p = 0.0315), and breed (p = 0.0231) of the dogs. The farm characteristics of the destination of the sewage (p = 0.0141) and garbage (p = 0.0018) also influenced the presence of parasites (Table 4). In this case, dogs of owners with higher education, younger animals (< 1 year), females, and crossbreds were more affected. Based on the odds ratio the farms group that sewage destination was not in septic tanks are 1.69 times more likely to have gastrointestinal parasitic infection in dogs, the females’ dogs are 1.39 times more likely to have gastrointestinal parasitic infection. On farms where sewage was kept in the environment or dumped into rivers, where garbage was stored in the environment or burned, and where dogs had access to the garbage, a higher occurrence of infected dogs was also observed (p < 0.05).

**Table 4:** Parameter estimates, odds rate lower and upper 95% confidence intervals and analysis of *Deviance* of the selected model^1^ for the occurrence of gastrointestinal parasitic infection in dogs, regardless of species.

**Variable**	**Estimate**	**Odds Ratio**	**Lower**	**Upper**	**Likelihood ratio**	**p-value**
Owner’s educational level	1.21	3.34	1.51	8.21	9.08	0.0026
Dog´s Age	-0.07	0.93	0.90	0.97	18.10	<0.0001
Gender	1.35	3.87	1.12	15.03	4.62	0.0315
Breed	1.39	4.03	1.20	15.96	5.16	0.0231
Sewage destination	1.69	5.40	1.40	23.4	6.02	0.0141
Garbage destination	-2.71	0.07	0.01	0.80	9.73	0.0018
Garbage access	-3.55	0.03	0.01	0.32	8.63	0.0033

1 Model selection was performed through a stepwise method using the Akaike information criterion (AIC) (Venables & Ripley, 2002)

## Discussion

One of the main economic activities in the southern region of Brazil is livestock rearing. This activity is also important for the municipality of Painel, Santa Catarina, where this study was conducted. Thus, several farmers were using dogs to help in their daily tasks, either in cattle management or for guarding these farms.

However, a high occurrence of dogs infected by important gastrointestinal parasites was observed, among which *Ancylostoma* spp. and *G. duodenalis* were the most prevalent, followed by *T. canis*, *T. vulpis*, *Entamoeba* spp., *Cryptosporidium* spp. and Taeniidae. This led us to reflect on the care that these dogs were receiving from their owners, as well as on the contamination of the study environment since the dogs live freely in the farms and have free access to all areas, then can defecate anywhere.

The parasitism observed in dogs in the rural area of the present study was close to the prevalence of infection found in a study conducted among dogs living in human homes in a region close to Painel, where *Ancylostoma* was also the most common parasite (Stalliviere et al., 2013), as well as in other studies on urban and rural prevalence (Bwalya et al., 2011; Torres-Chablé et al., 2015; Frizzo et al., 2016; Arruda et al., 2021). It is known that even with different parasitological methodologies, *Ancylostoma* is the genus most reported in dogs in Brazil (Labruna et al., 2006). This genus and *Toxocara* together are the main parasites responsible for environmental contamination, given that their eggs are eliminated in the feces of their hosts (Strube et al., 2013; Maleki et al., 2018).

In the present study, the molecular characterization of helminths and protozoa detected in dogs’ fecal samples was not carried out. As a specific parasite genetic analysis was not carried out, it is not possible to affirm the zoonotic potential of these etiological agents. Despite this, there is a possibility this can endanger the health of residents of the farms visited in the present study, remembering that one-third of them had children among the residents. It is known that humans suffer when L3 of *A. caninum* present in the soil enters the skin and causes cutaneous lesions ranging from local irritation to a cutaneous larva migrans syndrome. Besides this, accidental ingestion of *Toxocara* infective eggs from the soil or infective larvae in paratenic hosts may cause the visceral larva migrans and ocular larva migrans syndromes, though some infections are asymptomatic, severe symptoms are frequent in children, particularly toddlers (Morelli et al., 2021).

The protozoon *Cryptosporidium* spp. has often not been reported in prevalence studies, mainly due to diagnostic difficulty in stool samples, considering that its oocysts are difficult to visualize even using specific techniques for their diagnosis (Adeyemo et al., 2018). *Cryptosporidium* spp. was found in three dogs in the present study and has also been described infecting domestic dogs in Araçatuba, São Paulo (Bresciani et al., 2008), dogs at veterinary clinics in Canada (Uehlinger et al., 2013) and dogs in a rural area in Malaysia (Ngui et al., 2014). The zoonotic potential of this parasite, which is mainly disseminated through water, is favored in situations in which animals have access to humans’ water collection points, when the sewage is dumped into the river or discharged into the environment, as observed in the present study. Furthermore, the high resistance of these parasites’ oocysts means that they can remain viable even in water that receives treatment to ensure potability (Dreelin et al., 2014).

In addition, it is known that *Cryptosporidium* spp. is one of the main causes of diarrhea in calves. Adult animals with this parasite are considered to be sources of infection for the rest of the herd and contaminants of the environment (Vargas et al., 2014). This may be related to the occurrence of this parasite in the present study because dogs were being used for activities related to livestock management, where they had close and constant contact with cattle. Contamination of animals favors contamination of water catchment areas and water for human consumption because a single infected calf can eliminate 1 to 10 billion oocysts in its feces (Fayer et al., 2000).

Although *G. duodenalis* and *Cryptosporidium* spp. are different parasites, they have similar epizootiology and clinical manifestations (Morelli et al., 2021). *G. duodenalis* is a zoonotic protozoan classified into eight genotypes and is distributed worldwide (Fantinatti et al., 2018) and was found in 14.3% of sample feces in the present study. Dogs are frequently parasitized by zoonotic assemblages and cysts may be found in the feces of both healthy and diarrheic animals at similar percentages (reviewed by Morelli et al., 2021). These authors emphasize that more studies are necessary to ascertain the extent of the zoonotic transmission of *Giardia* since the cross-transmission cannot be proved just by the detection of the same assemblages in animal companions and humans. However, the zoonotic potential of this protozoan cannot be underestimated (Silva et al., 2022).

We found one dog that was infected by Taeniidae eggs. There is a possibility to be *Echinococcus* spp. since it was reported by the owners that the dogs have free access to carcasses and animal viscera. This would favor the development of the life cycle and dissemination of this parasite (Ingole et al., 2018). But we cannot claim for its certain identification of the *Echinococcus* spp. because through the use of morphologically-based microscopic techniques, we cannot distinguish these eggs from other Taeniidae.

Although *T. vulpis* was detected in feces samples in the present study, this report recalls the unresolved debate among researchers regarding the zoonotic potential of this parasite. Considering that its egg size differs from that of other species in that genus, such as *Trichuris trichiura* (Yoshikawa et al., 1989), there is a lack of clear data in the literature that could prove its relationship with infections in humans. Hence, currently, *T. vulpis* is still not included in all studies as a canine zoonotic parasite (Traversa, 2011).

However, in a study conducted in Malaysia, with molecular characterization of *Trichuris* species isolated from human and dog feces, it was shown that 1.3% of the parasites in human fecal samples were identified as *T. vulpis*, while in fecal samples from dogs, 56.8% and 43.2% were identified as *T. trichiura* and *T. vulpis*, respectively (Mohd-Shaharuddin et al., 2019). This finding implies that companion animals can be a reservoir and mechanical transmitter of *T. trichiura* infection in humans and also highlights the possible zoonotic potential of *T. vulpis*. It was also suggested through that study that cross-transmission between humans and hosts animals in a sympatric environment may be a source of infection in both hosts (Mohd-Shaharuddin et al., 2019).

Regarding the *Entamoeba* cysts that were found, although these were initially considered to be apathogenic parasites, diagnosing these species is important. There is difficulty in microscopically differentiating these commensal cosmopolitan colonizers of the intestines of humans and animals, especially *Entamoeba histolytica*, *Entamoeba dispar* and *Entamoeba moshkovskii* (morphologically indistinguishable), which are all species of veterinary medical importance (Dong et al., 2017).

In the present study, most dogs do not receive veterinarian care and rarely received antiparasitic treatment, even in dogs from owners with a high level of education. They were free to roam and had free access to animal remains and garbage, which was reflected in the significant associations with the occurrence of parasites that were found. Similar results were observed in a study conducted in Nigeria, in which most of the infected dogs were the ones that were allowed to move freely, had not been dewormed, and had received little or no veterinary care (Kamani et al., 2021). This situation puts public health at risk, as these animals can travel long distances and contaminate the environment with their feces (Kamani et al., 2021). The association of the owner’s education level with parasitism could be explained by the same motives related above. Even owners with higher education did not know how long ago the animals were dewormed (received at least one dose of anthelmintics in life) as well, not know if the treatment was effective, since fecal exams were never performed.

Young dogs (> 1 year) were significantly more infected by parasites, as observed in a study conducted in Nigeria (Kamani et al., 2021). In our study, there was a higher occurrence of gastrointestinal parasites in females, although some studies have reported that male dogs are more infected than females (Arruda et al., 2021; Kamani et al., 2021; Santos et al., 2021). The association of female dogs with enteric parasites can be related to the longer periods of immunosuppression that females go through, caused by gestations and puppies feeding, added to isolation during estrus, and poor diet, compared to males (Harvey et al., 2020).

When asked about what diseases dogs can transmit to humans, most participants reported that rabies was a source of infection from dogs to humans, which has commonly been reported in studies addressing this topic (Fontaine & Schantz, 1989; Bingham et al., 2010). These data are worrisome because they reflect a situation in which the dog’s owner is unconcerned about such diseases or does not know about other possible diseases, such as parasitic zoonoses, and thus takes on risk in relation to them.

Lack of knowledge about parasitosis may have been one of the causes of the owners’ negligence with regard to deworming their dogs. Insufficient administration of deworming drugs was the risk factor correlated with the occurrence of parasitic infection in the dog population in Alfenas-MG (Magalhães et al., 2020). In this regard, veterinarians are key influencers in improving dog owners’ perceptions about parasite control and also play a key role in promoting awareness of zoonoses transmitted within the community by pets (Nguyen et al., 2021).

Therefore, health education actions need to reach the population studied, and preventive measures need to be implemented, such as correct deworming of animals, adequate veterinary care and correct washing of hands and food. Dog owners and the general public in the study area need to be enlightened on the potential risks of parasitic zoonoses associated with dogs as well as the consequences of inadequate disposal of sewage and garbage, which could lead to environmental pollution that threatens human health.

Lastly, we conclude that rural dogs harbor gastrointestinal parasites, but their owners are unaware of the risks that these parasites can bring to human health.
